# Trends in the distribution of body mass index, waist circumference and prevalence of obesity among Taiwanese adults, 1993–2016

**DOI:** 10.1371/journal.pone.0274134

**Published:** 2022-09-09

**Authors:** Tzu-Jung Wong, Tsung Yu

**Affiliations:** 1 Department of Healthcare Information and Management, School of Health Technology, Ming Chuan University, Taoyuan, Taiwan; 2 Department of Academic Clinical Programme, National Dental Centre Singapore, Singapore, Singapore; 3 Department of Public Health, College of Medicine, National Cheng Kung University, Tainan, Taiwan; University of Coimbra: Universidade de Coimbra, PORTUGAL

## Abstract

**Background:**

Differences in the prevalence of general and abdominal obesity by subgroups such as age, sex, and education have been reported worldwide. Most studies in Taiwan regarding obesity prevalence were targeted at school-aged children or without further stratification by subgroups. Our aim was to examine the age-specific secular trend of body mass index (BMI), waist circumference (WC), and obesity prevalence stratified by sex, education and urbanization levels in Taiwanese adults.

**Methods:**

We used three waves of nationally representative population from the Nutrition and Health Survey in Taiwan (NAHSIT) 1993–1996 (n = 2 989), 2005–2008 (n = 2 495), and 2013–2016 (n = 2 880). The data included standardized measurement of body weight, height, and WC. We conducted a serial cross-sectional analysis among adults aged 20 years or above to examine the age-specific trends of BMI, WC, and the prevalence of underweight, overweight, general obesity, and abdominal obesity with stratification by sex, education, and urbanization levels.

**Results:**

The general obesity prevalence was 16%, 21%, and 20% and the abdominal obesity prevalence was 27%, 42%, and 47% in the 1993–1996, 2005–2008, and 2013–2016 surveys, respectively. The age-specific secular trend of BMI differed across subgroups; however, the trend of WC increased rapidly regardless of subgroups, except for women aged ≥60 years. The general obesity prevalence increased noticeably among men, younger- and middle-age adults with high school or higher education, middle- and older-age adults with lower than high school education, people <39 and ≥50 years of age residing in rural areas, and among those between 30 and 59 and ≥70 years of age residing in urban areas.

**Conclusions:**

Although the increasing trend of general obesity prevalence was levelling off among several subgroups, the abdominal obesity prevalence increased significantly and rapidly in Taiwan. Future research in developing effective weight and WC control interventions tailored to different subgroups is urgently needed.

## Introduction

Body mass index (BMI) is an anthropometric measure that is easy to obtain to categorize a person’s general adiposity. Although BMI has limitations when it is applied to individuals with abdominal obesity or high muscle mass, it is predictive of morbidity and mortality in the general population [1]. Meta-analysis using individual participant data shows that compared with people with BMI of 20 to 25 kg/m^2^, people with BMI below this range (underweight) or above this range (overweight) have slightly higher risk of all-cause death and the risk gets increasingly higher for obese people (BMI >30 kg/m^2^) [2]. These findings are generalizable to many populations worldwide.

However, since the BMI measure does not differentiate lean body mass from fat body mass and it does not reflect the distribution of body fat, researchers have suggested that other anthropometric measures such as waist circumference (WC) can also be used to predict morbidity and mortality [3]. The INTERHEART study, for instance, examined the association of BMI, WC, hip circumference, and waist-to-hip ratio with myocardial infarction; they found that the waist-to-hip ratio was most predictive of myocardial infarction [4]. It is thereby important to consider abdominal adiposity in addition to general adiposity while we are assessing the cardio-metabolic health of a population.

Understanding the secular trends (long-term trends) of general obesity and abdominal obesity would be informative for designing interventions and policies. Population-based research findings regarding the recent trends in the prevalence of obesity vary from country to country. In the US, the National Health and Nutrition Examination Survey showed an increasing linear trend in general obesity from 2005 to 2014 among women but not among men, after accounting for age, race/ethnicity, smoking, and education [5]. In Asia, the China Health and Nutrition Survey showed that from 1993 to 2015 the prevalence of overweight, general obesity and abdominal obesity increased markedly, particularly in men [6]. However, reports from Hong Kong and Korea indicated that trends in the prevalence of overweight/general obesity among women have levelled off or declined in recent years [7,8].

In Taiwan we found only one report discussing the secular trends in the prevalence of overweight and obesity using a population-based survey. Yeh and colleagues compared only two waves of the Nutrition and Health Survey in Taiwan (NAHSIT 1993–1996 and NAHSIT 2005–2008) and found that the prevalence of overweight, general obesity, and metabolic syndrome increased in both men and women [9]. Using the previous surveys and the recent NAHSIT 2013–2016, our goal was to (1) examine the trends in the distribution of BMI and waist circumferences in Taiwan, and (2) examine the trends in the prevalence of underweight, overweight, general obesity, and abdominal obesity in Taiwan. Our analysis of trends was age-specific; we stratified by sex, education and urbanization.

## Methods

The Nutrition and Health Survey in Taiwan (NAHSIT) was a government-sponsored national-representative cross-sectional health examination and nutrition survey conducted by the Ministry of Health and Welfare in Taiwan since 1992. The present study included three waves of the survey, NAHSIT 1993–1996, 2005–2008, and 2013–2016. The survey team in each wave used multi-staged stratified and clustered sampling to obtain a nationally representative sample of the Taiwanese non-institutionalized population; strata were formed based on the townships/districts in Taiwan and the probability proportional to population size method was used for sampling. The survey consisted of two components: (1) face-to-face household survey that was conducted at the participants’ homes, and (2) physical examination that was conducted at a temporary health examination station. The survey details have been reported elsewhere and all procedures of the study have been approved by the responsive ethics boards [10–13]. Datasets were obtained from the Health and Welfare Data Science Center, Ministry of Health and Welfare, Taiwan.

We included participants who were 20 to 79 years of age. There were 4 859, 4 404, and 5 377 participants aged 20 to 79 years in surveys conducted in 1993–1996, 2005–2008, and 2013–2016, respectively. Only about 60% of the participants attended the health examination. We included participants without missing data on weight or height values and excluded pregnant women. In total, our analysis had 62% (n = 2 989) participants of the 1993–1996 survey, 57% (n = 2 495) of the 2005–2008 survey, and 54% (n = 2 880) of the 2013–2016 survey.

Standardized equipment and techniques were used to measure WC in centimeters (cm), weight in kilograms (kg), and height in cm at the temporary health examination station. BMI was calculated as weight in kg divided by height in meters (m) squared. The BMI was categorized into four groups according to the recommendation of the World Health Organization for Asians: underweight (<18.5 kg/m^2^), normal weight (18.5– <23 kg/m^2^), overweight (23– <27.5 kg/m^2^), and obese (≥27.5 kg/m^2^). The abdominal obesity was defined as WC of > 90 cm for men and > 80 cm for women.

The demographic factors used in this study included age, sex, educational level, and urbanization level at interview. Participants were divided into six age groups with a 10-year cut-off: 20 to 29 years, 30 to 39 years, 40 to 49 years, 50 to 59 years, 60 to 69 years, and 70 to 79 years. Sex was categorized as female or male. The educational levels were grouped into two categories: less than high school and high school or above. For urbanization level, Liu in 2006 grouped the residential areas in Taiwan into seven categories (1–7), from the most urbanized to the least urbanized. The urbanization level of residential areas in this study was re-categorized into two groups: urban (categories 1–3) and rural (categories 4–7) areas.

Analyses were conducted separately for BMI, WC, abdominal obesity, general obesity, overweight, and underweight, stratified by sex, educational level, and urbanization level. For each stratification, we conducted age-group-specific analysis. The complex survey design and the differential probabilities of selection that resulted from non-response and non-coverage were taken into account with proper weighting when performing the analyses for the survey samples. Mean and standard error (SE) were reported for the continuous variables: BMI and WC. Simple linear regression was used to examine the secular trends in the mean value of BMI and WC over the three waves of the 4-year survey cycle. For categorical variables, including abdominal obesity, general obesity, overweight, and underweight, the percentage and SE were reported. Simple logistic regressions were used to examine the secular trends in the prevalence of abdominal obesity, general obesity, overweight, and underweight. To determine the statistical significance, a two-sided *P* value of less than 0.05 was considered. All analyses were conducted using Stata version 15 (StataCorp LLC, College Station, TX, USA) with the *svy* commands.

## Results

We summarize the prevalence of the selected characteristics for each wave of samples in Table 1. The distributions of age and sex were similar among survey waves. Men accounted for 47% (n = 1 407/2 989), 49% (n = 1 211/2 495), and 48% (n = 1 393/2 880) in the 1993–1996, 2005–2008 and 2013–2016 surveys, respectively. During the three survey waves conducted across 20 years, the prevalence of people who attained high school or above increased more than two fold, from 28% to 61%. The prevalence of people in the urban residential areas also increased from 36% to 48%.

**Table 1:** Characteristics of the sample.

	1993–1996 NAHSIT	2005–2008 NAHSIT	2013–2016 NAHSIT
**N**	2 989	2 495	2 880
**Men (%)**	1 407 (47)	1 211 (49)	1 393 (48)
**Age distribution (%)**			
** 20–29**	328 (11)	302 (12)	376 (13)
** 30–39**	500 (17)	269 (11)	366 (13)
** 40–49**	613 (21)	476 (19)	403 (14)
** 50–59**	609 (20)	491 (20)	588 (20)
** 60–69**	644 (22)	467 (19)	672 (23)
** 70–79**	295 (10)	490 (20)	475 (16)
**BMI in kg/m**^**2**^ **(SD)**	24.0 (3.8)	24.6 (4.0)	24.5 (4.1)
**Weight status (%)**			
** Underweight**	169 (6)	98 (4)	120 (4)
** Ideal**	1 083 (36)	814 (33)	1 026 (36)
** Overweight**	1 251 (42)	1 070 (43)	1 150 (40)
** Obesity**	486 (16)	513 (21)	584 (20)
**Waist circumference in cm (SD)**	78.9 (10.2)	83.2 (10.9)	84.8 (11.4)
**Abdominal obesity (%)**	816 (27)	1 057 (42)	1 355 (47)
**Attained high school or above education (%)**	842 (28)	1 162 (47)	1 757 (61)
**Urban area (%)**	1 087 (36)	1 155 (46)	1 392 (48)

BMI = body mass index, NAHSIT = Nutrition and Health Survey in Taiwan, SD = standard deviation.

Table 2 shows the trends in the BMI and WC mean values for each age group stratified by sex, educational levels and urbanization levels among Taiwanese adults for the three waves of the survey. Overall, people in the 1993–1996 survey had the lowest BMI mean values. Among men, BMI mean values ranged from 21.78 to 23.70 in the 1993–1996 survey, 23.15 to 24.96 in the 2005–2008 survey, and 23.43 to 25.28 in the 2013–2016 survey. Among women, BMI mean values ranged from 21.25 to 24.97 in the 1993–1996 survey, 21.72 to 25.30 in the 2005–2008 survey, and 22.26 to 24.73 in the 2013–2016 survey. We found there were increasing trends in the BMI mean values for most age groups in men except that the test for trend was marginally significant for men aged 50–59 (*p* = 0.058). However, the BMI mean values did not change much among survey waves for women across all age groups, as the tests for trend were not statistically significant.

**Table 2:** Trends in mean body mass index and waist circumference among Taiwanese adults: the NAHSIT 1993–2016.

	Body mass index (kg/m^2^)							Waist circumference (cm)						
	1993–1996		2005–2008		2013–2016		*p* for trend*	1993–1996		2005–2008		2013–2016		*p* for trend*
	n	Mean (SE)	n	Mean (SE)	n	Mean (SE)		n	Mean (SE)	n	Mean (SE)	n	Mean (SE)	
**Men**														
** **20–29	144	21.78 (0.34)	137	23.15 (0.41)	206	23.43 (0.48)	0.002	143	73.54 (0.89)	137	78.97 (1.19)	168	81.20 (1.09)	<0.001
** **30–39	209	23.20 (0.30)	121	24.73 (0.31)	198	25.16 (0.43)	<0.001	209	78.84 (1.02)	121	84.04 (0.87)	167	86.70 (1.14)	<0.001
** **40–49	291	23.43 (0.26)	221	24.46 (0.18)	218	25.19 (0.31)	<0.001	291	80.60 (0.53)	220	84.59 (0.52)	182	87.76 (0.87)	<0.001
** **50–59	270	23.70 (0.54)	239	24.96 (0.20)	303	24.86 (0.21)	0.058	270	82.85 (1.74)	237	86.96 (0.56)	282	88.35 (0.68)	0.003
** **60–69	345	23.67 (0.28)	229	24.68 (0.29)	328	25.28 (0.26)	<0.001	344	83.69 (0.95)	229	88.25 (0.84)	340	90.77 (0.75)	<0.001
** **70–79	148	22.87 (0.57)	264	24.01 (0.37)	234	24.52 (0.30)	0.013	148	81.42 (2.90)	263	87.00 (1.21)	236	90.34 (0.81)	0.005
**Women**														
** **20–29	184	21.25 (0.39)	165	21.72 (0.37)	170	22.26 (0.40)	0.078	183	67.26 (1.03)	165	72.02 (0.96)	202	74.95 (0.96)	<0.001
** **30–39	291	22.51 (0.31)	148	22.20 (0.37)	168	23.44 (0.53)	0.182	290	70.71 (0.55)	148	73.93 (0.98)	197	78.96 (1.30)	<0.001
** **40–49	322	23.72 (0.20)	255	23.82 (0.38)	185	23.17 (0.31)	0.169	322	73.33 (0.34)	255	76.72 (0.92)	217	78.23 (0.88)	<0.001
** **50–59	339	24.77 (0.19)	252	24.40 (0.29)	285	24.13 (0.25)	0.058	339	77.94 (0.47)	250	79.18 (0.78)	301	81.75 (0.66)	<0.001
** **60–69	299	24.97 (0.26)	238	25.25 (0.29)	344	24.40 (0.29)	0.112	299	81.29 (0.75)	238	83.27 (1.00)	321	82.95 (0.81)	0.159
** **70–79	147	24.88 (0.26)	226	25.30 (0.41)	241	24.73 (0.30)	0.703	147	81.04 (0.80)	223	86.61 (1.06)	229	84.67 (0.69)	0.001
**HS or above**														
** **20–29	223	21.15 (0.25)	267	22.45 (0.29)	353	22.72 (0.30)	<0.001	221	69.14 (0.62)	267	75.40 (0.73)	347	77.58 (0.75)	<0.001
** **30–39	250	22.26 (0.21)	223	23.47 (0.34)	332	24.10 (0.35)	<0.001	249	73.18 (0.45)	223	79.12 (0.94)	330	82.14 (0.96)	<0.001
** **40–49	188	23.53 (0.22)	291	23.94 (0.26)	324	23.86 (0.18)	0.355	188	77.57 (0.34)	290	80.63 (0.75)	322	82.00 (0.61)	<0.001
** **50–59	91	23.19 (0.37)	213	24.24 (0.28)	353	24.34 (0.28)	0.198	91	78.82 (1.25)	212	82.56 (0.96)	352	84.83 (0.89)	0.003
** **60–69	63	23.50 (0.58)	88	24.75 (0.42)	289	24.37 (0.29)	0.611	63	81.95 (1.56)	88	85.68 (1.38)	285	85.54 (0.94)	0.229
** **70–79	27	24.56 (1.01)	80	24.27 (0.59)	106	23.90 (0.56)	0.541	27	76.73 (9.02)	80	87.05 (1.38)	105	87.03 (1.31)	0.247
**Less than HS**														
** **20–29	105	22.37 (0.60)	35	21.47 (0.68)	23	24.50 (0.99)	0.666	105	73.66 (1.22)	35	73.86 (1.93)	23	82.99 (2.65)	0.047
** **30–39	249	23.44 (0.62)	46	23.62 (0.66)	34	25.66 (1.00)	0.182	249	76.38 (1.25)	46	78.85 (1.84)	34	86.17 (2.74)	0.004
** **40–49	423	23.60 (0.26)	185	24.58 (0.36)	79	25.25 (0.75)	0.008	423	76.73 (0.43)	185	80.81 (1.00)	77	85.43 (1.76)	<0.001
** **50–59	517	24.40 (0.39)	278	25.07 (0.32)	234	24.69 (0.20)	0.403	517	80.61 (0.98)	275	83.41 (0.83)	230	84.82 (0.66)	0.001
** **60–69	580	24.37 (0.23)	378	25.06 (0.22)	383	25.26 (0.30)	0.011	579	82.64 (0.72)	378	85.67 (0.67)	376	87.81 (0.83)	<0.001
** **70–79	268	23.72 (0.37)	409	24.73 (0.26)	368	24.84 (0.22)	0.012	268	81.82 (1.25)	405	86.74 (0.71)	359	87.51 (0.66)	<0.001
**Urban**														
** **20–29	138	21.69 (0.31)	131	22.31 (0.41)	187	22.38 (0.36)	0.113	138	70.65 (0.59)	131	75.06 (0.90)	183	76.94 (0.98)	<0.001
** **30–39	188	22.82 (0.32)	132	23.36 (0.31)	179	24.35 (0.45)	0.007	188	74.86 (0.56)	132	79.02 (0.79)	177	82.68 (1.20)	<0.001
** **40–49	257	23.50 (0.22)	240	23.88 (0.26)	194	24.09 (0.26)	0.075	257	77.17 (0.23)	240	79.84 (0.70)	193	82.58 (0.85)	<0.001
** **50–59	204	24.44 (0.36)	227	24.23 (0.23)	291	24.11 (0.26)	0.462	204	80.70 (0.95)	226	82.22 (0.78)	289	84.31 (0.82)	0.004
** **60–69	200	24.51 (0.14)	226	25.02 (0.19)	327	24.63 (0.31)	0.770	200	83.41 (0.49)	226	85.73 (0.82)	321	86.10 (0.97)	0.019
** **70–79	100	24.08 (0.27)	199	24.65 (0.36)	214	24.64 (0.32)	0.159	100	81.46 (2.07)	197	86.60 (1.05)	209	87.88 (0.80)	0.004
**Rural**														
** **20–29	190	21.06 (0.48)	171	22.53 (0.36)	189	23.55 (0.45)	<0.001	188	70.11 (1.46)	171	75.73 (1.27)	187	79.33 (1.06)	<0.001
** **30–39	312	22.95 (0.29)	137	23.78 (0.60)	187	23.94 (0.33)	0.028	311	74.57 (1.56)	137	79.23 (1.71)	187	81.96 (0.80)	<0.001
** **40–49	356	23.81 (0.24)	236	24.69 (0.38)	209	24.12 (0.30)	0.483	356	76.58 (0.99)	235	82.49 (0.70)	206	82.55 (0.80)	<0.001
** **50–59	405	23.76 (0.53)	264	25.52 (0.34)	297	25.22 (0.22)	0.049	405	79.57 (1.87)	261	84.48 (1.15)	294	85.87 (0.65)	0.003
** **60–69	444	23.81 (0.53)	241	24.89 (0.31)	345	25.17 (0.24)	0.021	443	80.89 (1.85)	241	85.53 (1.05)	340	87.76 (0.83)	0.001
** **70–79	195	23.33 (0.59)	291	24.63 (0.35)	261	24.62 (0.30)	0.073	195	80.85 (2.25)	289	87.15 (0.96)	256	86.71 (0.92)	0.032

*Linear trends in the mean body mass index and waist circumference were tested using linear regression model.

HS = high school, NAHSIT = Nutrition and Health Survey in Taiwan, SE = standard error.

Among people who attained high school or above, there were significant increasing trends in the BMI mean values for those aged 20–29 (*p*<0.001) and 30–39 (*p*<0.001). Among people with less than a high school degree, there were increasing trends in the BMI mean values for those aged 40–49 (*p* = 0.008), 60–69 (*p* = 0.011) and 70–79 (*p* = 0.012). People aged 20–29, 30–39, 50–59 and 60–69 in rural areas had significant increasing trends in the BMI mean values (*p*<0.001, *p* = 0.028, *p* = 0.049 and *p* = 0.021, respectively); in the urban areas, only people aged 30–39 had an increasing trend in the BMI mean values (*p* = 0.007).

The trends in the WC mean values appeared to be more consistently increasing than the trends in the BMI mean values among various defined subgroups. We found statistically significant increasing trends in the WC mean values across all age groups stratified by sex, educational levels and urbanization levels. The test for trend was not significant in only three groups: women aged 60–69 (*p* = 0.159) and people aged 60–69 and 70–79 with high school or above education (*p* = 0.229 and *p* = 0.247, respectively).

Table 3 presents the trends in the prevalence of underweight and overweight for each age group stratified by sex, educational levels and urbanization levels. Overall, people aged 20–29 had the highest prevalence of underweight. The prevalence of underweight decreased from 1993–1996 to 2005–2008 but increased slightly from 2005–2008 to 2013–2016. We found statistically significant decreasing trends in the prevalence of underweight among people aged 60–69 except for the subgroup of women. People aged 20–29 residing in the rural areas and people aged 70–79 with lower than high school education or residing in the urban areas also had statistically significant decreasing trends (*p* = 0.043, *p* = 0.023 and *p* = 0.033, respectively).

**Table 3:** Trends in prevalence of underweight and overweight among Taiwanese adults: the NAHSIT 1993–2016.

	Underweight							Overweight						
	1993–1996		2005–2008		2013–2016		*p* for trend*	1993–1996		2005–2008		2013–2016		*p* for trend*
	n	% (SE)	n	% (SE)	n	% (SE)		n	% (SE)	n	% (SE)	n	% (SE)	
**Men**														
** **20–29	144	17 (6)	137	5 (2)	170	10 (3)	0.187	144	34 (5)	137	47 (6)	170	46 (5)	0.061
** **30–39	209	3 (2)	121	3 (2)	168	4 (2)	0.785	209	49 (5)	121	69 (6)	168	64 (4)	0.009
** **40–49	291	5 (2)	221	2 (1)	185	1 (1)	0.099	291	59 (5)	221	70 (3)	185	73 (4)	0.039
** **50–59	270	5 (2)	239	1 (1)	285	2 (1)	0.176	270	52 (8)	239	73 (4)	285	74 (4)	0.012
** **60–69	345	5 (2)	229	0 (0)	344	0 (0)	<0.001	345	55 (2)	229	69 (3)	344	75 (3)	<0.001
** **70–79	148	10 (5)	264	4 (1)	241	2 (1)	0.050	148	42 (8)	264	59 (3)	241	64 (4)	0.020
**Women**														
** **20–29	184	24 (4)	165	18 (4)	206	17 (3)	0.152	184	21 (5)	165	26 (5)	206	31 (4)	0.141
** **30–39	291	6 (3)	148	6 (3)	198	9 (4)	0.589	291	31 (3)	148	30 (5)	198	37 (4)	0.396
** **40–49	322	1 (1)	255	5 (2)	218	2 (1)	0.333	322	59 (4)	255	50 (5)	218	45 (4)	0.018
** **50–59	339	2 (1)	252	1 (0)	303	7 (2)	0.061	339	68 (3)	252	58 (5)	303	58 (3)	0.048
** **60–69	299	3 (1)	238	2 (1)	328	2 (1)	0.389	299	67 (4)	238	76 (3)	328	58 (5)	0.092
** **70–79	147	4 (2)	226	3 (1)	234	1 (1)	0.095	147	66 (4)	226	69 (4)	234	65 (4)	0.814
**HS or above**														
** **20–29	223	21 (4)	267	12 (2)	353	14 (3)	0.104	223	27 (3)	267	37 (4)	353	38 (3)	0.006
** **30–39	250	6 (2)	223	5 (2)	332	7 (2)	0.846	250	34 (3)	223	51 (5)	332	47 (3)	0.010
** **40–49	188	1 (1)	291	4 (2)	324	2 (1)	0.986	188	57 (6)	291	59 (3)	324	57 (3)	0.903
** **50–59	91	10 (5)	213	1 (1)	353	5 (2)	0.670	91	54 (9)	213	62 (5)	353	64 (4)	0.458
** **60–69	63	4 (2)	88	3 (3)	289	1 (1)	0.017	63	60 (10)	88	64 (6)	289	64 (4)	0.836
** **70–79	27	4 (3)	80	2 (1)	106	2 (2)	0.580	27	58 (13)	80	68 (6)	106	59 (6)	0.906
**Less than HS**														
** **20–29	105	20 (7)	35	8 (8)	23	0 (0)	0.189	105	29 (7)	35	18 (7)	23	34 (14)	0.645
** **30–39	249	3 (1)	46	0 (0)	34	2 (1)	0.255	249	45 (6)	46	41 (5)	34	71 (10)	0.251
** **40–49	423	4 (1)	185	2 (1)	79	2 (1)	0.225	423	61 (3)	185	62 (5)	79	63 (7)	0.736
** **50–59	517	3 (1)	278	1 (1)	234	3 (2)	0.998	517	61 (4)	278	68 (4)	234	69 (3)	0.111
** **60–69	580	4 (1)	378	1 (0)	383	1 (1)	0.027	580	61 (3)	378	75 (3)	383	68 (4)	0.048
** **70–79	268	8 (3)	409	3 (1)	368	2 (1)	0.023	268	53 (6)	409	63 (3)	368	66 (3)	0.044
**Urban**														
** **20–29	138	18 (5)	131	12 (3)	187	14 (4)	0.431	138	30 (3)	131	34 (5)	187	34 (4)	0.457
** **30–39	188	5 (2)	132	3 (2)	179	8 (3)	0.659	188	38 (3)	132	49 (5)	179	49 (4)	0.017
** **40–49	257	4 (1)	240	5 (2)	194	1 (1)	0.228	257	57 (3)	240	58 (3)	194	58 (4)	0.921
** **50–59	204	5 (1)	227	1 (1)	291	6 (2)	0.623	204	63 (3)	227	62 (4)	291	62 (3)	0.832
** **60–69	200	4 (1)	226	2 (1)	327	1 (1)	0.033	200	64 (4)	226	72 (2)	327	64 (4)	0.957
** **70–79	100	6 (3)	199	2 (1)	214	1 (1)	0.033	100	54 (4)	199	62 (3)	214	68 (4)	0.029
**Rural**														
** **20–29	190	27 (7)	171	12 (5)	189	12 (3)	0.043	190	19 (8)	171	39 (7)	189	46 (4)	0.016
** **30–39	312	2 (1)	137	6 (3)	187	4 (1)	0.162	312	45 (4)	137	51 (8)	187	50 (4)	0.360
** **40–49	356	1 (0)	236	1 (1)	209	3 (2)	0.098	356	65 (5)	236	64 (3)	209	58 (3)	0.171
** **50–59	405	0 (0)	264	1 (1)	297	2 (1)	0.096	405	53 (8)	264	72 (6)	297	73 (3)	0.042
** **60–69	444	3 (1)	241	0 (0)	345	0 (0)	0.010	444	55 (2)	241	72 (6)	345	69 (4)	0.004
** **70–79	195	9 (6)	291	5 (1)	261	2 (1)	0.149	195	51 (13)	291	67 (5)	261	60 (4)	0.545

*Linear trends in prevalence of underweight and overweight were tested using logistic regression model.

HS = high school, NAHSIT = Nutrition and Health Survey in Taiwan, SE = standard error.

The trends of overweight prevalence were increasing among men across all age groups except for people aged 20–29. But among women, the prevalence of overweight decreased significantly in those aged 40–49 and 50–59 (*p* = 0.018 and *p* = 0.048, respectively). Statistically significant increasing trends of overweight were found in several subgroups: people with high school or higher education aged 20–29 and 30–39, people with lower than high school education aged 60–69 and 70–79, people in the urban areas aged 30–39 and 70–79, and people in the rural areas aged 20–29, 50–59, and 60–69.

We present the trends in the prevalence of general obesity and abdominal obesity for each age group stratified by sex, educational levels and urbanization levels in Table 4. The prevalence of general obesity consistently increased among young and middle-aged populations but not among older populations without regard to the defined subgroups. For instance, significant increasing trends were observed among people aged 30–39 in all subgroups. The most rapid increase in the prevalence of general obesity was observed among those with lower than high school education aged 30–39 (9% to 35%, *p* = 0.006) and 40–49 (13% to 38%, *p* = 0.003).

**Table 4:** Trends in prevalence of general and abdominal obesity among Taiwanese adults: the NAHSIT 1993–2016.

	General obesity							Abdominal obesity						
	1993–1996		2005–2008		2013–2016		*p* for trend*	1993–1996		2005–2008		2013–2016		*p* for trend*
	n	% (SE)	n	% (SE)	n	% (SE)		n	% (SE)	n	% (SE)	n	% (SE)	
**Men**														
** **20–29	144	5 (2)	137	14 (3)	170	13 (3)	0.011	143	4 (2)	137	17 (4)	168	15 (3)	0.003
** **30–39	209	8 (2)	121	24 (5)	168	27 (4)	<0.001	209	7 (3)	121	27 (4)	167	30 (4)	<0.001
** **40–49	291	10 (1)	221	8 (2)	185	25 (4)	0.001	291	9 (2)	220	18 (3)	182	34 (4)	<0.001
** **50–59	270	10 (4)	239	21 (3)	285	20 (3)	0.067	270	25 (6)	237	34 (4)	282	45 (4)	0.01
** **60–69	345	15 (3)	229	15 (4)	344	20 (4)	0.226	344	22 (3)	229	43 (6)	340	53 (4)	<0.001
** **70–79	148	12 (4)	264	14 (4)	241	17 (3)	0.313	148	25 (8)	263	39 (5)	236	52 (4)	0.006
**Women**														
** **20–29	184	7 (3)	165	10 (2)	206	13 (3)	0.204	183	7 (3)	165	16 (3)	202	22 (4)	0.014
** **30–39	291	5 (2)	148	10 (3)	198	20 (4)	0.003	290	9 (2)	148	20 (4)	197	36 (5)	<0.001
** **40–49	322	12 (2)	255	16 (4)	218	12 (2)	0.931	322	18 (3)	255	35 (4)	217	42 (5)	<0.001
** **50–59	339	22 (2)	252	18 (3)	303	19 (3)	0.605	339	38 (2)	250	46 (4)	301	55 (3)	<0.001
** **60–69	299	22 (4)	238	25 (4)	328	18 (3)	0.427	299	52 (5)	238	61 (5)	321	62 (4)	0.135
** **70–79	147	25 (4)	226	28 (4)	234	15 (3)	0.079	147	52 (5)	223	77 (4)	229	76 (4)	<0.001
**HS or above**														
** **20–29	223	3 (2)	267	12 (2)	353	13 (2)	0.001	221	3 (2)	267	16 (3)	347	19 (3)	<0.001
** **30–39	250	4 (2)	223	17 (3)	332	22 (3)	<0.001	249	3 (2)	223	23 (3)	330	31 (3)	<0.001
** **40–49	188	8 (1)	291	10 (2)	324	13 (2)	0.039	188	8 (2)	290	24 (3)	322	36 (4)	<0.001
** **50–59	91	6 (3)	213	15 (3)	353	17 (3)	0.267	91	20 (6)	212	33 (4)	352	50 (4)	<0.001
** **60–69	63	8 (4)	88	16 (5)	289	15 (3)	0.539	63	26 (5)	88	36 (5)	285	50 (4)	0.001
** **70–79	27	24 (14)	80	10 (5)	106	9 (4)	0.237	27	27 (9)	80	44 (7)	105	48 (6)	0.095
**Less than HS**														
** **20–29	105	11 (6)	35	14 (6)	23	12 (6)	0.862	105	12 (6)	35	16 (6)	23	14 (7)	0.631
** **30–39	249	9 (3)	46	19 (7)	34	35 (13)	0.006	249	13 (4)	46	27 (6)	34	53 (13)	0.002
** **40–49	423	13 (2)	185	16 (4)	79	38 (8)	0.003	423	16 (3)	185	32 (5)	77	49 (8)	<0.001
** **50–59	517	18 (3)	278	23 (3)	234	25 (3)	0.092	517	34 (3)	275	47 (4)	230	50 (4)	0.001
** **60–69	580	19 (2)	378	21 (3)	383	24 (3)	0.256	579	37 (3)	378	57 (4)	376	64 (3)	<0.001
** **70–79	268	17 (2)	409	24 (3)	368	19 (3)	0.497	268	39 (6)	405	61 (4)	359	69 (3)	<0.001
**Urban**														
** **20–29	138	5 (2)	131	13 (3)	187	11 (3)	0.033	138	5 (2)	131	16 (2)	183	15 (4)	0.007
** **30–39	188	7 (1)	132	15 (3)	179	24 (4)	<0.001	188	8 (2)	132	22 (3)	177	33 (4)	<0.001
** **40–49	257	12 (1)	240	11 (3)	194	16 (3)	0.260	257	14 (2)	240	26 (3)	193	39 (5)	<0.001
** **50–59	204	18 (3)	227	15 (2)	291	18 (3)	0.954	204	34 (3)	226	36 (4)	289	49 (4)	0.003
** **60–69	200	20 (2)	226	22 (2)	327	18 (4)	0.719	200	39 (3)	226	52 (5)	321	58 (5)	0.001
** **70–79	100	19 (4)	199	22 (5)	214	16 (3)	0.684	100	41 (4)	197	56 (5)	209	67 (4)	<0.001
**Rural**														
** **20–29	190	8 (6)	171	9 (2)	189	17 (3)	0.343	188	8 (6)	171	17 (5)	187	24 (3)	0.091
** **30–39	312	7 (4)	137	23 (7)	187	20 (4)	0.046	311	8 (3)	137	27 (5)	187	33 (5)	<0.001
** **40–49	356	6 (1)	236	15 (3)	209	20 (3)	0.001	356	10 (3)	235	27 (4)	206	37 (4)	<0.001
** **50–59	405	11 (13)	264	28 (3)	297	24 (3)	0.044	405	26 (8)	261	47 (4)	294	54 (4)	0.006
** **60–69	444	15 (16)	241	16 (4)	345	21 (3)	0.329	443	30 (9)	241	52 (5)	340	56 (3)	0.011
** **70–79	195	16 (2)	291	20 (3)	261	17 (3)	0.949	195	31 (8)	289	60 (5)	256	61 (4)	0.007

*Linear trends in prevalence of general and abdominal obesity were tested using logistic regression model.

HS = high school, NAHSIT = Nutrition and Health Survey in Taiwan, SE = standard error.

The increasing trends in the prevalence of abdominal obesity were similar to the trends in WC mean values. We found statistically significant increasing trends in the prevalence of abdominal obesity across all age groups stratified by sex, educational levels and urbanization levels. The test for trend was not significant in only three groups: women aged 60–69 (*p* = 0.135) and people aged 20–29 with lower than high school education and residing in rural areas (*p* = 0.631 and *p* = 0.091, respectively). We observed the highest prevalence of abdominal obesity (77%) among women aged 70–79 in the 2005–2008 survey.

## Discussion

Our study aimed at examining the age-specific trends in the following: (1) distribution of BMI and WC and (2) prevalence of underweight, overweight, general obesity and abdominal obesity in Taiwan, stratified by sex, education, and urbanization. Our findings suggest that, although the overall trend of mean BMI and WC has levelled off, the prevalence of abdominal obesity has increased nearly two-fold from 1993 to 2016.

After conducting further age-specific analysis, the overall trends in the mean BMI and WC as well as in the prevalence of overweight, general obesity, and abdominal obesity could be briefly summarized as follows. A markedly increasing trend in the prevalence of general obesity was found among men; most results were not significant among women. Increasing trends were found particularly in younger- and middle-age adults with high school or higher education and in middle- and older-age adults with lower than high school education. Increasing trends were also found among people aged <39 and ≥50 years residing in rural areas and among those aged between 30 and 59 and ≥70 years residing in urban areas. We also found that there is a decreasing trend in the prevalence of underweight, particularly among those aged 60–79 years.

The Non-Communicable Disease Risk Factor Collaboration (NCD-RisC) reported that the overall prevalence of general obesity continues to be high and estimated that the prevalence is expected to increase up to 18% in men and 21% in women worldwide by 2025[14]. In addition, the prevalence of abdominal obesity in the US has increased steadily up to more than 50% since 2004 [15]. Our findings are consistent with studies that indicate the overall trend of general and abdominal obesity has been increasing progressively over time [16,17].

Studies have also indicated that the prevalence of general and abdominal obesity was generally higher among women than among men, regardless of age [17]. We had similar patterns in the 1993–1996 and the 2005–2008 surveys. In the 2013–2016 survey, however, the prevalence of general obesity among women was the same or lower than among men for most age groups. The overall prevalence of abdominal obesity has increased over time regardless of sex in countries such as the US and China, but the findings in Korea showed a decreasing trend among women from 1998 to 2014 [6,8]. In our study, the prevalence of abdominal obesity increased rapidly regardless of sex, yet the upward trend was flattening among women aged ≥ 60 years. Although women in most populations worldwide are more likely to be obese than men due to a variety of sociocultural and socioeconomic reasons and their reproductive role [18,19], such a trend was not found in the recent (2013–2016) survey in Taiwan. Studies have highlighted how social norms and thin-ideal media exposure may influence the obesity prevalence, especially among women [20,21]. Women in East Asian countries such as Taiwan and South Korea were found to be more prone to the societal pressures of thin-ideal body image [22]. There is an increasing trend of media consumption and internet use in Taiwan, and internet users have increased from about 9.5 million in 2003 to 18.8 million in 2016 [23–25]. Women are at a greater risk of being exposed to the thin-ideal image and social pressure to be thin, resulting in the increase of body dissatisfaction, misperception of the ideal body, disordered eating behaviors, and unhealthy weight control, especially among female adolescents [22,26,27].

The impact of educational levels on obesity was inconsistent in previous studies. Studies have underscored that the differential measurements of educational attainment could capture different underlying constructs, such as years of education, degree of education, intelligence, and illiteracy or not, which could influence the association between educational attainment and health outcomes [28–31]. A systematic review on educational attainment and obesity concluded that a positive relationship between obesity and educational level was seen more in low-income countries, whereas a reverse relationship was more common in high-income countries [6,30,32,33]. The relationship can also be moderated by sex, as among women; the association between educational attainment and obesity is more consistent and further away from the null than among men, for which it has more variations [30]. Our results showed that the prevalence of general obesity and abdominal obesity among people with a lower than high school education was higher than people with a high school or higher education. This relationship between education and obesity may be affected by how the educational attainment was measured and other factors such as poverty levels and occupations [30,34–36], which requires further investigation.

Studies have also reported differed trends of general obesity and abdominal obesity stratified by the residential areas. The results from the National Nutrition Survey 1976–95 in Japan showed that regardless of age and sex, except for women aged 50 years and above, the prevalence of general obesity was generally higher in small towns than in cities or in metropolitan areas [37]. Similar patterns were was found in the US, that the obesity (BMI≥30) and severe obesity (BMI ≥40) prevalence was higher in non-metropolitan statistical areas (MSAs; large: ≥1 million population) and in medium or small MSAs than in large MSAs during 2001 to 2016, regardless of sex [38]. However, the findings from China showed that people who resided in urban areas had a higher prevalence of general obesity from 1993 to 2008 than people in rural areas, but the prevalence was higher in the rural areas than the urban areas from 2009 to 2015 [6,39]. People residing in the urban areas also had a higher prevalence of abdominal obesity than those in rural areas, although the differences have decreased [6,39]. Our results were consistent with previous studies [6,37,39]. General obesity was more prevalent in rural areas, particularly in the 2013–2016 survey, while abdominal obesity was more prevalent only in rural areas in the 2005–2008 survey. Our results also suggested that general and abdominal obesity prevalence increased dramatically from 2008 to 2013 in rural areas and were in line with studies that examined the trends worldwide and in other countries [37,38,40]. The BMI findings in rural areas were persistently higher than urban areas in high-income and industrialized countries [40]. The increased prevalence of mechanized work in rural areas may result in decreased levels of physical activities and further result in an increased risk of obesity [41]. In addition, the lack of nutrition knowledge and the attitudes in rural areas may increase the likelihood of malnutrition or excessive consumption of low-quality calories, and could lead to increased prevalence of general and abdominal obesity [40,42].

This study has several limitations. First, we were not able to examine BMI and WC trajectories at the individual level because the data were from cross-sectional surveys instead of longitudinal studies. Second, although the multi-staged stratified and clustered sampling approach was used to collect data, selection bias could not be avoided. People who did not show up for the health examination and who had missing anthropometric data were not included in this study. Third, we only included people aged 20 years and above in our analysis. Since the general pattern of BMI and WC changes for children and adolescents may be quite different from adults, further studies are needed if our research question is also targeting younger populations. Last, factors such as body fat, muscle mass, caloric intake, and physical activities were not discussed in this study. The trend of BMI is strongly associated with body fat and muscle mass [43]. As we were not able to distinguish whether the high prevalence of general obesity was the result of high body fat or high muscle mass, there may be misclassification of being “fat,” especially among men [43].

Despite the limitations, our study has some strengths. To our knowledge, this is one of the first studies in Taiwan to examine multiple waves of nationally representative data—NAHSIT 1993–1996, 2005–2008, and 2013–2016—to understand the pattern of WC and BMI. Instead of using the self-reported weight and height, which could be biased as people tend to overreport their height and underreport their weight, we used data collected by standardized equipment and techniques [44]. Based on the standardized measurements, our results provided a more precise estimation of the prevalence of underweight, overweight, general and abdominal obesity. Most importantly, our study also provided detailed information regarding the stratified age-specific prevalence of abdominal obesity, which has been widely reported to be associated with all-cause cardiovascular disease, and cancer mortality, even among people with normal BMI [45–47].

### Conclusions

The three waves of nationally representative surveys of adults in Taiwan demonstrated that the pattern of general and abdominal obesity varied by subgroup populations. Although the general obesity prevalence has decreased in certain age groups among women, the prevalence of abdominal obesity was increasing, indicating that women with normal BMI may have abdominal obesity. Besides, the WC increased rapidly over time, and the estimates for most of the age-specific prevalence of abdominal obesity also increased regardless of sex, educational attainment, and urbanization levels. The results of our study emphasized the importance of developing and executing interventions to flatten and decrease the general and abdominal obesity prevalence and informed regarding which subgroup should be the future research target.

## References

[1] Pischon T, Boeing H, Hoffmann K, Bergmann M, Schulze MB, Overvad K et al. General and abdominal adiposity and risk of death in Europe. *New England Journal of Medicine* 2008; 359(20): 2105–2120. doi: 10.1056/NEJMoa0801891 19005195

[2] Global BMI Mortality Collaboration, Di Angelantonio E, Bhupathiraju Sh N, Wormser D, Gao P, Kaptoge S et al. Body-mass index and all-cause mortality: individual-participant-data meta-analysis of 239 prospective studies in four continents. *Lancet* 2016; 388(10046): 776–86. doi: 10.1016/S0140-6736(16)30175-1 27423262 PMC4995441

[3] Huxley R, Mendis S, Zheleznyakov E, Reddy S, Chan J. Body mass index, waist circumference and waist:hip ratio as predictors of cardiovascular risk—a review of the literature. *Eur J Clin Nutr* 2010; 64(1): 16–22. doi: 10.1038/ejcn.2009.68 19654593

[4] Yusuf S, Hawken S, Ounpuu S, Bautista L, Franzosi MG, Commerford P et al. Obesity and the risk of myocardial infarction in 27,000 participants from 52 countries: a case-control study. *Lancet* 2005; 366(9497): 1640–9. doi: 10.1016/S0140-6736(05)67663-5 16271645

[5] Flegal KM, Kruszon-Moran D, Carroll MD, Fryar CD, Ogden CL. Trends in Obesity Among Adults in the United States, 2005 to 2014. *JAMA* 2016; 315(21): 2284–91. doi: 10.1001/jama.2016.6458 27272580 PMC11197437

[6] Ma S, Xi B, Yang L, Sun J, Zhao M, Bovet P. Trends in the prevalence of overweight, obesity, and abdominal obesity among Chinese adults between 1993 and 2015. Int J Obes (Lond) 2021; 45(2): 427–437. 33037330 10.1038/s41366-020-00698-x

[7] Ko GT, Tang JS, Chan JC. Worsening trend of central obesity despite stable or declining body mass index in Hong Kong Chinese between 1996 and 2005. *Eur J Clin Nutr* 2010; 64(5): 549–52. doi: 10.1038/ejcn.2010.49 20332802

[8] Shin HY, Kang HT. Recent trends in the prevalence of underweight, overweight, and obesity in Korean adults: The Korean National Health and Nutrition Examination Survey from 1998 to 2014. *J Epidemiol* 2017; 27(9): 413–419. doi: 10.1016/j.je.2016.08.014 28420559 PMC5565760

[9] Yeh CJ, Chang HY, Pan WH. Time trend of obesity, the metabolic syndrome and related dietary pattern in Taiwan: from NAHSIT 1993–1996 to NAHSIT 2005–2008. *Asia Pac J Clin Nutr* 2011; 20(2): 292–300. 21669598

[10] Lin YC, Yen LL, Chen SY, Kao MD, Tzeng MS, Huang PC et al. Prevalence of overweight and obesity and its associated factors: findings from National Nutrition and Health Survey in Taiwan, 1993–1996. *Prev Med* 2003; 37(3): 233–41. doi: 10.1016/s0091-7435(03)00119-1 12914829

[11] Tu SH, Chen C, Hsieh YT, Chang HY, Yeh CJ, Lin YC et al. Design and sample characteristics of the 2005–2008 Nutrition and Health Survey in Taiwan. *Asia Pac J Clin Nutr* 2011; 20(2): 225–37. 21669592

[12] Pan W-H, Kao M-D, Tzeng M-S, Yen L-L, Hung Y-T, Li L-A et al. Nutrition and Health Survey in Taiwan (NAHSIT) 1993–1996: Design, Contents, and Operations. *Journal of the Chinese Nutrition Society* 1999; 24(1): 1–10.

[13] Pan W-H. The Nutrition and Health Survey in Taiwan (NAHSIT) 2013–-2016 Report. (In Chinese). 2019. https://www.hpa.gov.tw/EngPages/Detail.aspx?nodeid=1077&pid=6201. Accessed 8 Jan 2021.

[14] NCD Risk Factor Collaboration (NCD-RisC). Trends in adult body-mass index in 200 countries from 1975 to 2014: a pooled analysis of 1698 population-based measurement studies with 19.2 million participants. *Lancet* 2016; 387(10026): 1377–1396. 27115820 10.1016/S0140-6736(16)30054-X PMC7615134

[15] Centers for Disease Control and Prevention. Chronic Kidney Disease Surveillance System—United States. 2021. https://nccd.cdc.gov/CKD/. Accessed 8 Jan 2021.

[16] Wong MCS, Huang J, Wang J, Chan PSF, Lok V, Chen X et al. Global, regional and time-trend prevalence of central obesity: a systematic review and meta-analysis of 13.2 million subjects. *Eur J Epidemiol* 2020; 35(7): 673–683. doi: 10.1007/s10654-020-00650-3 32448986 PMC7387368

[17] GBD 2015 Obesity Collaborators, Afshin A, Forouzanfar MH, Reitsma MB, Sur P, Estep K et al. Health Effects of Overweight and Obesity in 195 Countries over 25 Years. *N Engl J Med* 2017; 377(1): 13–27. doi: 10.1056/NEJMoa1614362 28604169 PMC5477817

[18] Finucane MM, Stevens GA, Cowan MJ, Danaei G, Lin JK, Paciorek CJ et al. National, regional, and global trends in body-mass index since 1980: systematic analysis of health examination surveys and epidemiological studies with 960 country-years and 9.1 million participants. *Lancet* 2011; 377(9765): 557–67. 21295846 10.1016/S0140-6736(10)62037-5 PMC4472365

[19] Garawi F, Devries K, Thorogood N, Uauy R. Global differences between women and men in the prevalence of obesity: is there an association with gender inequality? *Eur J Clin Nutr* 2014; 68(10): 1101–6. doi: 10.1038/ejcn.2014.86 24918120

[20] Shoham DA, Hammond R, Rahmandad H, Wang Y, Hovmand P. Modeling social norms and social influence in obesity. *Curr Epidemiol Rep* 2015; 2(1): 71–79. doi: 10.1007/s40471-014-0032-2 26576335 PMC4643315

[21] Crielaard L, Dutta P, Quax R, Nicolaou M, Merabet N, Stronks K et al. Social norms and obesity prevalence: From cohort to system dynamics models. *Obes Rev* 2020; 21(9): e13044. doi: 10.1111/obr.13044 32400030 PMC7507199

[22] Noh JW, Kwon YD, Yang Y, Cheon J, Kim J. Relationship between body image and weight status in east Asian countries: comparison between South Korea and Taiwan. *BMC Public Health* 2018; 18(1): 814. doi: 10.1186/s12889-018-5738-5 29970058 PMC6029392

[23] Mingoia J, Hutchinson AD, Wilson C, Gleaves DH. The Relationship between Social Networking Site Use and the Internalization of a Thin Ideal in Females: A Meta-Analytic Review. *Front Psychol* 2017; 8: 1351. doi: 10.3389/fpsyg.2017.01351 28824519 PMC5545754

[24] Taiwan network information center. Survey on Broadband and Wireless Usage in Taiwan. 2003. https://twnic.tw/stat_n.php. Accessed 8 Jan 2021.

[25] Taiwan network information center. 2016 Summary Report: Wireless Internet Usage in Taiwan. 2016. https://twnic.tw/stat_n.php. Accessed 8 Jan 2021.

[26] Chang FC, Lee CM, Chen PH, Chiu CH, Pan YC, Huang TF. Association of thin-ideal media exposure, body dissatisfaction and disordered eating behaviors among adolescents in Taiwan. Eat Behav 2013; 14(3): 382–5. doi: 10.1016/j.eatbeh.2013.05.002 23910785

[27] Shih MY, Kubo C. Body shape preference and body satisfaction of Taiwanese and Japanese female college students. *Psychiatry Res* 2005; 133(2–3): 263–71. doi: 10.1016/j.psychres.2004.10.008 15741001

[28] Yu ZB, Han SP, Cao XG, Guo XR. Intelligence in relation to obesity: a systematic review and meta-analysis. *Obes Rev* 2010; 11(9): 656–70. doi: 10.1111/j.1467-789X.2009.00656.x 19780990

[29] Deary IJ, Johnson W. Intelligence and education: causal perceptions drive analytic processes and therefore conclusions. *Int J Epidemiol* 2010; 39(5): 1362–9. doi: 10.1093/ije/dyq072 20504860

[30] Cohen AK, Rai M, Rehkopf DH, Abrams B. Educational attainment and obesity: a systematic review. *Obes Rev* 2013; 14(12): 989–1005. doi: 10.1111/obr.12062 23889851 PMC3902051

[31] Backlund E, Sorlie PD, Johnson NJ. A comparison of the relationships of education and income with mortality: the National Longitudinal Mortality Study. *Soc Sci Med* 1999; 49(10): 1373–84. doi: 10.1016/s0277-9536(99)00209-9 10509827

[32] Hajian-Tilaki KO, Heidari B. Association of educational level with risk of obesity and abdominal obesity in Iranian adults. *J Public Health (Oxf)* 2010; 32(2): 202–9. doi: 10.1093/pubmed/fdp083 19689983

[33] Hsieh TH, Lee JJ, Yu EW, Hu HY, Lin SY, Ho CY. Association between obesity and education level among the elderly in Taipei, Taiwan between 2013 and 2015: a cross-sectional study. *Sci Rep* 2020; 10(1): 20285. doi: 10.1038/s41598-020-77306-5 33219305 PMC7680111

[34] Lahti-Koski M, Vartiainen E, Mannisto S, Pietinen P. Age, education and occupation as determinants of trends in body mass index in Finland from 1982 to 1997. *Int J Obes Relat Metab Disord* 2000; 24(12): 1669–76. doi: 10.1038/sj.ijo.0801437 11126222

[35] Galobardes B, Morabia A, Bernstein MS. The differential effect of education and occupation on body mass and overweight in a sample of working people of the general population. *Ann Epidemiol* 2000; 10(8): 532–7. doi: 10.1016/s1047-2797(00)00075-2 11118933

[36] Yang YC, Walsh CE, Johnson MP, Belsky DW, Reason M, Curran P et al. Life-course trajectories of body mass index from adolescence to old age: Racial and educational disparities. *Proc Natl Acad Sci U S A* 2021; 118(17). doi: 10.1073/pnas.2020167118 33875595 PMC8092468

[37] Yoshiike N, Seino F, Tajima S, Arai Y, Kawano M, Furuhata T et al. Twenty-year changes in the prevalence of overweight in Japanese adults: the National Nutrition Survey 1976–95. *Obes Rev* 2002; 3(3): 183–90. doi: 10.1046/j.1467-789x.2002.00070.x 12164470

[38] Hales CM, Fryar CD, Carroll MD, Freedman DS, Aoki Y, Ogden CL. Differences in Obesity Prevalence by Demographic Characteristics and Urbanization Level Among Adults in the United States, 2013–2016. *JAMA* 2018; 319(23): 2419–2429. doi: 10.1001/jama.2018.7270 29922829 PMC6583043

[39] Xi B, Liang Y, He T, Reilly KH, Hu Y, Wang Q et al. Secular trends in the prevalence of general and abdominal obesity among Chinese adults, 1993–2009. *Obes Rev* 2012; 13(3): 287–96. 10.1111/j.1467-789X.2011.00944.x PMC3276709 22034908

[40] NCD Risk Factor Collaboration (NCD-RisC). Rising rural body-mass index is the main driver of the global obesity epidemic in adults. *Nature* 2019; 569(7755): 260–264. doi: 10.1038/s41586-019-1171-x 31068725 PMC6784868

[41] Pickett W, King N, Lawson J, Dosman JA, Trask C, Brison RJ et al. Farmers, mechanized work, and links to obesity. *Prev Med* 2015; 70: 59–63. doi: 10.1016/j.ypmed.2014.11.012 25448840

[42] Lin W, Hang CM, Yang HC, Hung MH. 2005–2008 Nutrition and Health Survey in Taiwan: the nutrition knowledge, attitude and behavior of 19–64 year old adults. *Asia Pac J Clin Nutr* 2011; 20(2): 309–18. 21669600

[43] Gallagher D, Visser M, Sepulveda D, Pierson RN, Harris T, Heymsfield SB. How useful is body mass index for comparison of body fatness across age, sex, and ethnic groups? *Am J Epidemiol* 1996; 143(3): 228–39. doi: 10.1093/oxfordjournals.aje.a008733 8561156

[44] Merrill RM, Richardson JS. Validity of self-reported height, weight, and body mass index: findings from the National Health and Nutrition Examination Survey, 2001–2006. *Prev Chronic Dis* 2009; 6(4): A121. PMC2774635 19754997

[45] Zhang C, Rexrode KM, van Dam RM, Li TY, Hu FB. Abdominal obesity and the risk of all-cause, cardiovascular, and cancer mortality: sixteen years of follow-up in US women. *Circulation* 2008; 117(13): 1658–67. doi: 10.1161/CIRCULATIONAHA.107.739714 18362231

[46] Kim HY, Kim JK, Shin GG, Han JA, Kim JW. Association between Abdominal Obesity and Cardiovascular Risk Factors in Adults with Normal Body Mass Index: Based on the Sixth Korea National Health and Nutrition Examination Survey. * Obes Metab Syndr* 2019; 28(4): 262–270. doi: 10.7570/jomes.2019.28.4.262 31909369 PMC6939698

[47] Despres JP, Lemieux I, Bergeron J, Pibarot P, Mathieu P, Larose E et al. Abdominal obesity and the metabolic syndrome: contribution to global cardiometabolic risk. *Arterioscler Thromb Vasc Biol* 2008; 28(6): 1039–49. doi: 10.1161/ATVBAHA.107.159228 18356555

