# Supplementary material for: Trends in the distribution of body mass index, waist circumference and prevalence of obesity among Taiwanese adults, 1993–2016
Source: PLoS One. 2022 Sep 9;17(9):e0274134. doi: 10.1371/journal.pone.0274134 (PMC9462812; doi:10.1371/journal.pone.0274134)
Supplement: Attachment — Submitted filename: Response to Reviewers.docx [file pone.0274134.s001.docx]

**Response to Reviewers**

1. Reviewer #1: The manuscript is suitable with the Plos One article type. However, minor changes should be performed in order to fit all the guidelines. Although the aim, authors refer to obesity prevalence it does not have an impact on the title that is used in the article. Keywords should be clarified and focusing the content and study aim (they seem general and not specific for the topic).

***Response:***

*We made changes to the title (page 1 in the manuscript with tracked changes) and keywords (page 4) accordingly.*

2. Definition for secular trend should appear in the introduction because it’s a term repeated on the document.

***Response:***

*In this study secular trend is the long-term trend, and we made changes to the Introduction section accordingly (line 20 on page 6).*

3. Which reference is used for general obesity and abdominal obesity? And why sometimes is used just obesity and others general obesity?

***Response:***

*We updated reference 1 (N Engl J Med. 2008 Nov 13;359(20):2105-20), where there are definitions for general and abdominal obesity. We clarified these two terms throughout the manuscript.*

4. At methods, Stata 15 reference seems incomplete.

***Response:***

*We edited the reference accordingly (line 92 on page 9).*

5. Reviewer #2: PLOS ONE

Submission: PONE-D-22-05300

An increase in mean body mass index or prevalence of obesity may be accompanied by changes in the population BMI distribution. This study aimed to examine how the distributions of BMI and waist circumference have changed in Taiwan over a 23-year interval (1998-2014).

I highlight:

1. The number of included data;

2. Study relevance;

3. Overall english.

***Response:***

*We are not fully clear about where we should revise specifically. The number of data can be found in the Methods section (line 60 on page 8). Study relevance are clarified in the Introduction section. We had the manuscript edited by a professional English editor.*

6. Some details should be improved:

1. Data analysis;

2. Methods details.

***Response:***

*We edited the Methods section accordingly to clarify the data analysis.*

7. I suggest authors examine the distributional changes (over time) in BMI (or WC), using square regressions for all individuals and subgroups (stratified by sex, age, educational level, and residence area), including graphs or figures in the manuscript according to previous related works:

Kim, S., Subramanian, S. V., Oh, J., & Razak, F. (2018). Trends in the distribution of body mass index and waist circumference among South Korean adults, 1998-2014. Eur J Clin Nutr, 72(2), 198-206. <https://doi.org/10.1038/s41430-017-0024-7>

***Response:***

*We thank for the suggestions. We in fact did use linear regression and logistic regression when we were testing for the trend effects (see lines 85-91 on page 9).*
